# Identification, Characterization, and Genome Analysis of Two Novel Temperate *Pseudomonas protegens* Phages PseuP_222 and PseuP_224

**DOI:** 10.3390/microorganisms11061456

**Published:** 2023-05-31

**Authors:** Vera Morozova, Yuliya Kozlova, Artem Tikunov, Igor Babkin, Tatyana Ushakova, Alevtina Bardasheva, Ghadeer Jdeed, Elena Zhirakovskaya, Alina Mogileva, Sergei Netesov, Nina Tikunova

**Affiliations:** 1 Institute of Chemical Biology and Fundamental Medicine Siberian Branch of Russian Academy of Sciences, Novosibirsk 630090, Russia; morozova@niboch.nsc.ru (V.M.);; 2 Faculty of Natural Sciences, Novosibirsk State University, Novosibirsk 630090, Russiasvn15@hotmail.com (S.N.)

**Keywords:** *Pseudomonas protegens*, environmental *Pseudomonas* phage, lambdoid phage, comparative genomics, proteomic analysis

## Abstract

Two novel *P. protegens* bacteriophages PseuP_222 and Pseu_224 and their host *P. protegens* CEMTC 4060 were isolated from the same sample (Inya river, Siberia). Both phages have siphovirus morphology and belong to lambdoid phages. Comparative genome analysis revealed a low nucleotide and amino acid sequence similarity of PseuP_222 and PseuP_224 between themselves, and between them and other lambdoid phages. Bioinformatics analysis indicated that PseuP_222 and PseuP_224 are members of a genetically diverse group of phages of environmental *Pseudomonas* spp.; this group is distant from a large group of *P. aeruginosa* phages. In phylogenetic trees, the positioning of the terminase large subunits, major capsid proteins, tail tape measure proteins, and CI-like repressors of PseuP_222 and PseuP_224 were remote and changed relative to those of the *Escherichia* lambda phage and lambdoid phages of *Pseudomonas* spp. However, the nucleoid-associated protein NdpA/YejK and P5-like structural protein from both phages showed high similarity and were not found in lambda phage and other lambdoid phages of *Pseudomonas* spp. Substantial divergences of the PseuP_222 and PseuP_224 genomes and proteomes indicated that the evolutionary history of these phages was mostly independent and they probably began to use one host only recently.

## 1. Introduction

*Pseudomonas protegens* is a member of the diverse and complex genus *Pseudomonas*, which contains widespread Gram-negative bacteria. Fluorescent *P. protegens* bacteria have been separated from *Pseudomonas fluorescens* for their ability to produce the antimicrobial compound pyoluterin in addition to 2,4-diacetylphloroglucinol [Ramette, 2011]. Multilocus sequence analysis based on 16S rRNA, *rpoB*, *rpoD*, and *gyrB* phylogenies has shown independent clustering of *P. protegens* strains [1]. Detailed genetic characterization has placed *P. protegens* in the *P. fluorescens* group [2,3,4]. *P. protegens* bacteria are distributed overall and can be found in a wide variety of environmental niches, especially rhizosphere soil. *P. protegens* is a typical soil microorganism with an extremely versatile metabolism and can be isolated from roots of various plant species. This bacterium is a strict aerobe, which grows at temperatures between 4 °C and 36 °C [5,6]. Bacteria of this species contribute to soil improvement by suppressing fungal and bacterial pathogens with antimicrobials and they have been extensively studied for possible biological control of plant diseases [7,8]. In addition, *P. protegens* can colonize plant roots and herbivorous insects; some strains effectively use both eukaryotic organisms [9,10,11,12,13]. When invading such diverse niches, *P. protegens* bacteria encounter various microbial communities and deploy many metabolites, toxins, and bacteriocins, allowing them to counteract other competitors in the colonized environment [14,15,16]. Notably, some contenders are phylogenetically close *Pseudomonas* strains; therefore, *P. protegens* have to produce narrow-spectrum tools, as close relatives are usually resistant to broad-spectrum weapons [5,17].

Bacteriophages, like phage-like bacteriocins (tailocins), usually exhibit a narrow specificity and are used by their producers to destroy closely related competitors [5,6].

The NCBI GeneBank database (https://www.ncbi.nlm.nih.gov, accessed on 16 May 2023), contains 1084 pseudomonas phage complete genomes; of them, more than 750 belong to *Pseudomonas aeruginosa* phages, 140 to *Pseudomonas syringae* phages, 60 to *P. fluorescens* phages, and 40 to *Pseudomonas putida* phages. In addition, several genomes of phages specific to some other members of the *Pseudomonas* genus—*P. agarici*, *P. fremontii*, *P. chlororaphis P. savastanoi*, and *P. stutzeri*—are presented in the GeneBank. Although *P. protegens* have been intensively studied for their plant-beneficial and entomopathogenic activities, only one specific bacteriophage has been reported [18]. Lytic *P. protegens* phage ΦGP100 has been isolated from rhizosphere soil in Switzerland and found to be specific to *P. protegens* CHA0 and related strains of the same species [18]. This phage has podovirus morphology and a 50,547-bp genome with 76 predicted open reading frames, and belongs to the *Zobellviridae* family [19].

In this study, we describe two novel temperate *P. protegens* phages, PseuP_222 and PseuP_224, infecting the same host strain *P. protegens* CEMTC 4060. Both phages and the host were derived from the same water sample from the river Inya, in the Novosibirsk region (Western Siberia). These phages were characterized in terms of their biological properties, and analysis of their genomes indicated substantial differences between themselves, and between them and other known phages of *Pseudomonas* spp.

## 2. Materials and Methods

### 2.1. Host Strain Isolation and Culture Conditions

The isolation procedure of the *P. protegens* strain was similar to that described in detail previously [20]. Briefly, a drop of water from the river Inya, in the Novosibirsk region, was diluted in sterile phosphate-buffered saline, pH 7.5, and aliquots of different dilutions were poured onto nutrient agar (Microgen, Obolensk, Russia). Plates were incubated overnight at 25 °C and the obtained various colonies were independently passaged three or more times. A 16S rRNA gene fragment (1308 bp) was sequenced to confirm *P. protegens* identification as described previously [21]. In addition, PCR fragments of the *rpoD*, *gyrB*, and *fdxA* genes were obtained and sequenced according to [22]. The primers used for PCR are given in Supplementary Table S1. The strain *P. protegens* CEMTC 4060 was deposited in the Collection of Extremophilic Microorganisms and Type Cultures (CEMTC) of the Institute of Chemical Biology and Fundamental Medicine SB RAS, Novosibirsk.

In order to induce prophages, an exponentially growing culture of the host strain *P. protegens* CEMTC 4060 (an optical density of 0.4 at 600 nm) was treated with mitomycin C, 0.5 μg/mL (Sigma-Aldrich, St. Louis, MO, USA) and then incubated with shaking for 24 h. Growth or possible cell lysis of the culture was screened by hourly measurements of OD600. In addition, a fresh layer of *P. protegens* CEMTC 4060 in the top agar with 0.8% bacteriological agar (OXOID, Basingstoke, UK) was exposed to ultraviolet irradiation for 5, 10, and 15 s. The plates were incubated for 42 h and the appearance of plaques was regularly checked. These assays were performed with three replicates.

### 2.2. Isolation and Propagation of the PseuP_222 and PseuP_224 Phages

Both phages were selected from the same water sample, which was previously used for *P. protegens* CEMTC 4060 isolation, using the procedure described previously [20], with a single modification. The plates were incubated overnight at 25 °C, and two types of plaques were observed. Considering that different types of plaques were produced by different phages, each type of plaque was suspended in sterile PBS to extract phage particles. To clone the phages, tenfold dilutions of each phage suspension were independently spotted on a fresh layer of *P. protegens* CEMTC 4060. The obtained single plaques of different types were used for subsequent independent phage extraction. The cloning procedure was repeated five times for each type of plaques.

The morphology of plaques formed by the PseuP_222 and PseuP_224 phages on a layer of *P. protegens* CEMTC 4060 was determined using the double agar overlay method [23]. Plaques were examined after overnight incubation at 25 °C.

Both phages PseuP_222 and PseuP_224 were propagated by infecting *P. protegens* CEMTC 4060 cultivated at OD_600_ = 0.6 in nutrient broth (NB; Condalab, Madrid, Spain). Multiplicity of infection (MOI) was 0.1. In both cases, infected host culture was incubated at 25 °C for 30 min without shaking and then with shaking. The total cultivation time until cell lysis was different for both phages.

### 2.3. Phage Particle Morphology

In order to visualize phage particles, transmission electron microscopy (TEM) was used. Suspensions of each phage (10^9^ pfu/mL) were individually adsorbed for 1 min on a formvar-coated copper grid. The grids were contrasted with uranyl acetate for 5–7 s and examined with a TEM JEM 1400 (JEOL, Tokyo, Japan). A side-mounted Veleta digital camera (Olympus SIS, Münster, Germany) was used to obtain digital pictures.

### 2.4. Biological Properties and Host Range Study

In order to evaluate the biological properties of PseuP_222 and PseuP_224 phages, all experiments were repeated twice, and each experiment had three replicates. In all cases, cultures were incubated at 25 °C. Experiments on phage adsorption rate and burst size were carried out according to [24,25] with our modifications [20]. The lytic activity of the PseuP_222 and PseuP_224 phages was determined in a procedure described previously [26] with our modifications. Individual phages PseuP_222 (10^7^ pfu/mL), PseuP_224 (10^7^ pfu/mL), or their mixture (5 × 10^6^ pfu/mL for each phage) were added to an exponentially growing culture of *P. protegens* CEMTC 4060 (10^8^ CFU/mL). The obtained mixtures were incubated with shaking at 25 °C. Aliquots were taken from each mixture every 30 min, diluted, and spread on the nutrient agar plates. After overnight incubation at 25 °C, the emerging colonies were counted, and bacterial killing curves were built based on the obtained numbers. To estimate the host range for the PseuP_222 and PseuP_224 phages, a convenient spot-assay method [27] was carried out. Strains belonging to 28 *Pseudomonas* species were used for the assay.

### 2.5. Phage DNA Purification and Complete Genome Sequencing

Phage DNA was purified by the method described previously [28]. Briefly, phage particles were concentrated by polyethylene glycol 6000 (PEG 6000; AppliChem, Darmstadt, Germany). The pellet was dissolved in STM-buffer (10 mM NaCl; 50 mM Tris-HCl, pH 8.0; 10 mM MgCl_2_) and treated with RNase and DNase (Thermo Fisher Scientific, Waltham, MA, USA) for 1 h at 37 °C. Then, EDTA, proteinase K (Thermo Fisher Scientific, Waltham, MA, USA), and SDS were added to the phage suspension to final concentrations of 20 mM, 100–200 mkg/mL, and 0.5%, respectively. After the mixture was incubated for 3 h at 55 °C, DNA was extracted by phenol/chloroform with subsequent ethanol precipitation.

Preparing of paired-end libraries by the Nextera DNA Sample Preparation Kit (Illumina, Inc., San Diego, CA, USA) and subsequent sequencing using the MiSeq Benchtop Sequencer and MiSeq Reagent Kit v.1 (both Illumina Inc., San Diego, CA, USA) were carried out as usual. The SPAdes genome assembler v.3.15.2 (http://cab.spbu.ru/software/spades, accessed on 15 November 2021) was used to de novo assemble complete genome sequences of the studied phages. PseuP_222 and PseuP_224 genome sequences were deposited in the GenBank database (accession numbers OP626800 and OP795451, respectively).

### 2.6. Genome Analysis

Rapid Annotation Subsystem Technology (RAST) v.2.0 (https://rast.nmpdr.org, accessed on 16 September 2022) was used to annotate the putative open reading frames (ORFs). Then, the results of annotation were manually verified by checking all of the predicted proteins against the NCBI GenBank protein database (https://www.ncbi.nlm.nih.gov, accessed on 19 September 2022). BLASTX and DELTA-BLAST algorithms were used for comparing the polypeptides encoded by the predicted ORFs with sequences deposited in the GenBank database. InterProScan and HHPred software were used to analyse the predicted ORFs, encoding hypothetical proteins and ORFs without homology with the sequences deposited in the GenBank database [29,30]. Virulence factors and the antibiotic-resistance genes were searched using the Virulence Factor database (http://www.mgc.ac.cn/VFs, accessed on 30 September 2022) and Antibiotic Resistance Gene database (https://card.mcmaster.ca/rgi, accessed on 30 September 2022), respectively.

Alignment of complete genome sequences of bacteriophages was performed in the MAFFT program (https://mafft.cbrc.jp accessed on 13 April 2023). The starting points of the studied sequences were manually changed in accordance with the PseuP_222 sequence based on dot-plot analysis in the UGENE program [31]. The intergenomic similarity was calculated in the BioEdit 7.2.5 program [32]. A sequence identity matrix was calculated using the Virus Intergenomic Distance Calculator (VIRIDIC (http://rhea.icbm.uni-oldenburg.de/VIRIDIC accessed on 13 April 2023) and ViPTree server. PHASTER software (https://phaster.ca, accessed on 10 October 2022) was used for the search of prophage sequences in *Pseudomonas* genomes.

### 2.7. Proteome Analysis

The Viral Proteomic Tree (ViPTree) server (https://www.genome.jp/viptree, accessed 3 October 2022) was used to perform a comparative proteomic phylogenetic analysis. For the analysis, appropriate phages were extracted from the NCBI GenBank database and Virus-Host database (https://www.genome.jp/virushostdb, accessed 3 October 2022).

### 2.8. Phylogenetic Analysis

To search for the corresponding protein sequences, the BLASTP program was used (https://blast.ncbi.nlm.nih.gov/ accessed on 13 April 2023). The selected amino acid sequences were aligned in the T-Coffee program using the M-Coffee algorithm (https://tcoffee.crg.eu accessed on 13 April 2023). All phylogenetic trees were constructed using the maximum likelihood (ML) method based on the LG model in MEGA 7.0 [33]. All phylogenetic trees were midpoint-rooted.

## 3. Results

### 3.1. Bacterial Host Isolation

The *Pseudomonas protegens* CEMTC 4060 strain was isolated from a water sample taken from the Inya River (55°06′13.07″ N 83°30′49.26″ E, Novosibirsk region, Russia), which is a right bank tributary of the big Siberian River Ob. The isolated strain was identified as *P. protegens* by sequencing of 16S rRNA, *rpo*D, *gyr*B, and *fdx*A gene fragments; sequences were deposited in the GenBank database under accession numbers ON838113, OP820967, OP820965, and OP820966, respectively. The strain *P. protegens* CEMTC 4060 was mesophilic and was able to grow at temperature of 25 °C and above. The strain was deposited in CEMTC.

As *P. protegens* CEMTC 4060 was used as the host for the studied phages, experiments on the prophage induction were carried out. After ten passages and upon exposure to stress conditions (mitomycin C or ultraviolet irradiation), no plaques were revealed. Indeed, not all prophages can be induced by such methods, and other stress conditions for bacteria may be required.

### 3.2. PseuP_222 and PseuP_224 Plaque and Phage Morphology

Phages PseuP_222 and PseuP_224, as well as the host strain *P. protegens* CEMTC 4060, were isolated from the same water sample. When a drop of the sterilized water sample was spotted onto a fresh layer of *P. protegens* CEMTC 4060, two types of plaques were observed: small cloudy plaques with a diameter of 0.5–1 mm and large clear plaques with a diameter of 2 mm. Small plaques were surrounded by a weak translucent halo; the PseuP_222 phage was subsequently isolated from one of them. The PseuP_224 phage was isolated from a clear plaque.

**Figure 1:**
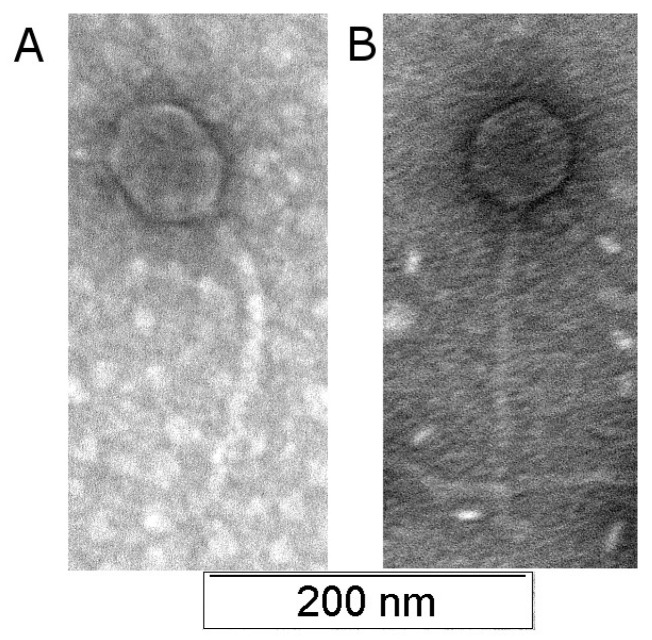
Visualization of the PseuP_222 and PseuP_224 plaques (**A**,**B**). Electron micrograph of PseuP_222 and PseuP_224 AerP_220 phage particles negative staining with 1% uranyl acetate.

Electron microscopy showed that PseuP_222 and PseuP_224 phages have elongated icosahedral and icosahedral heads with diameters of ~60 nm and ~80 nm, respectively. Their heads are connected to long flexible tails of approximately 200 nm in length for both phages (Figure 1). The morphology of both phages corresponds to siphovirus morphotype [34].

### 3.3. Biological Properties and Host Range

**Figure 2:**
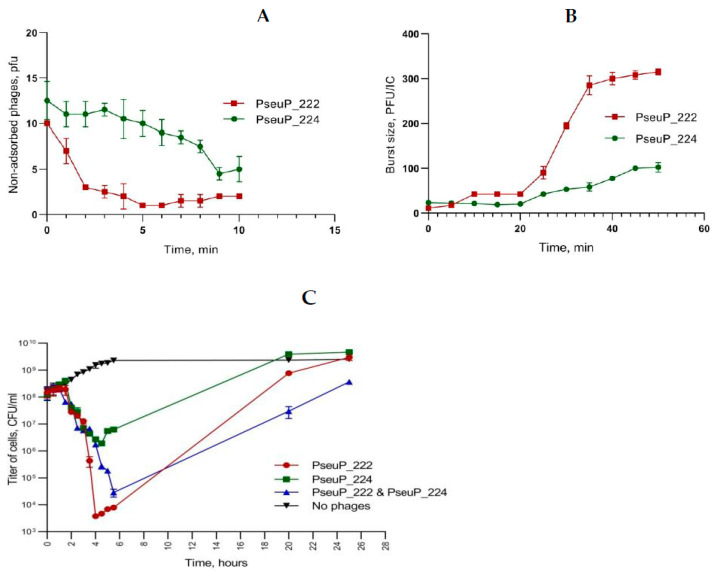
Adsorption of phages PseuP_222 and PseuP_224 to *P. protegens* CEMTC 4060 (**A**); burst sizes and latent periods of phages PseuP_222 and PseuP_224 on the same host (**B**); multistep bacterial killing curves of individual phages PseuP_222 and PseuP_224 and their combination on the same host (**C**).

Adsorption (Figure 2A) and one-step growth assays (Figure 2B) were performed to evaluate the biological properties of phages PseuP_222 and PseuP_224. The obtained results indicated that the lytic activity of the phages differed. PseuP_222 adsorbed to the host *P. protegens* CEMTC 4060 relatively quickly and 90% of phage particles attached to cells after five min. The adsorption time for PseuP_224 was 9 min and only 50% of phage particles bound during the period (Figure 2A).

One-step growth curves for phages PseuP_222 and PseuP_224 indicated that latent periods were approximately 30 and 45 min, respectively. The burst size for PseuP_222 and PseuP_224 differed and was ~300 and ~100 phage particles per infected cell, respectively (Figure 2B). The multistep bacterial killing curves (Figure 2C) showed that the amount of living bacteria decreased dramatically by five orders of magnitude in 4 h after infection with PseuP_222, whereas a decrease of only three orders of magnitude was detected in 4.5 h after infection with PseuP_224. Notably, the mixture of the phages had a lower lytic rate than that of PseuP_222, and the minimum of living cells occurred at 5.5 h. This result indicates the possible competition of phages PseuP_222 and PseuP_224 for the cellular receptor. However, the lytic activity was more prolonged for the mixture than for the individual phages, which reflects a synergistic effect of species-specific phage cocktails (Figure 2C).

A host range assay was carried out using 95 *Pseudomonas* strains deposited in CEMTC. Of them, 76 strains were environmental (including *P. protegens,* n = 9 and *Pseudomonas aeruginosa,* n = 7), whereas 19 strains were isolated from clinical samples (*P. protegens,* n = 1, and *P. aeruginosa,* n = 18) (Supplementary Table S2). Among ten *P. protegens* strains, seven strains were isolated from various natural water reservoirs and rivers, two from insects, and one from a clinical sample (smear from a site with dry gangrene). Both PseuP_222 and PseuP_224 had narrow host specificity. The *P. protegens* strain CEMTC 4060 is the only known susceptible strain for PseuP_222, whereas PseuP_224 can infect *P. protegens* CEMTC 4060 and one more *P. protegens* strain CEMTC 5980, which was isolated from a nearby (~50 km) Ob reservoir (Supplementary Table S2). The PseuP_224 phage produced clear plaques on the *P. protegens* CEMTC 5980; however, the titer was approximately ten times lower than that for *P. protegens* CEMTC 4060 (Supplementary Table S2). Notably, *P. protegens* strains CEMTC 4060 and CEMTC 5980 were isolated respectively from the Inya River and an Ob reservoir, both belonging to the Ob River basin. However, the Inya River flows into the Ob River below the Ob Reservoir and it would be difficult for *P. protegens* CEMTC 5980 to get into the Inya River, where both phages were found. Other tested *P. protegens* strains, as well as environmental and clinical *Pseudomonas* strains (Supplementary Table S2), were not susceptible to infection by PseuP_222 and PseuP_224. Therefore, phages PseuP_222 and PseuP_224 occupy the same niche, use one host strain, *P. protegens* CEMTC 4060, for replication, and do not infect *Pseudomonas hydrolytica* and *Pseudomonas khazarica* strains isolated from the same location (Supplementary Table S2). Nevertheless, only ten available *P. protegens* strains were screened, so *P. protegens* strains sensitive to these phages might be found.

### 3.4. PseuP_222 and PseuP_224 Genome Characteristics and Comparative Analysis

The PseuP_222 and PseuP_224 genomes substantially differ in size: 56,717 bp and 43,181 bp, respectively. The number of putative ORFs also varies: 81 and 60 ORFs were recorded in the genomes, respectively (Supplementary Datas S1 and S2). In the PseuP_222 genome, 48 ORFs encode proteins with predicted functions, whereas products of 33 ORFs are hypothetical proteins (Supplementary Data S1). In the PseuP_224 genome, predicted functions are assigned to 37 ORFs, while 23 ORFs encode polypeptides with unknown functions (Supplementary Data S2). Genes encoding DNA polymerase, DNA-dependent RNA polymerase, and virulence factors, as well as antibiotic-resistance genes were not found in the PseuP_222 and PseuP_224 genomes.

**Figure 3:**
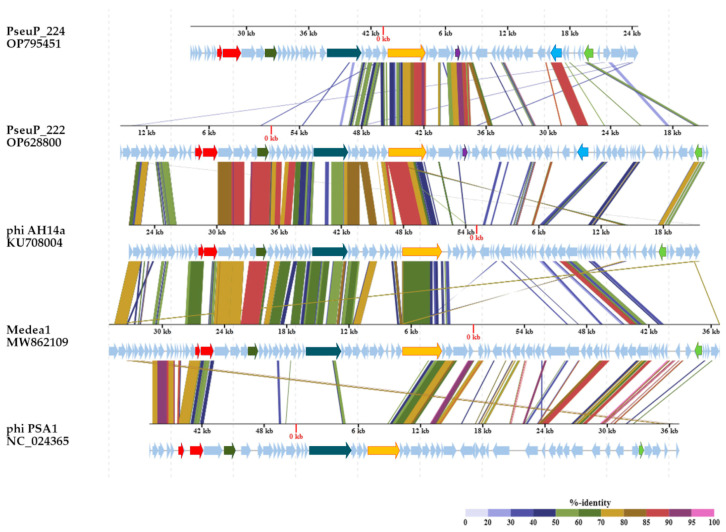
Comparative genome alignment of the PseuP_222 and PseuP_224 phages and the most similar *Pseudomonas* phages phiAH14a (KU708004.1), Medea1 (MW862109), and phiPSA1 (NC_024365). Analysis was performed using VipTree software. Genes encoding small and large terminase subunits are marked with red; genes of capsid proteins are marked with olive colour; genes of tail tape measure proteins are marked with dark cyan; genes of tail tip proteins J are marked with yellow; genes of P5-like proteins are marked with lilac; genes of NdpA/YejK are marked with light blue; genes encoding CI-like repressors are marked with light green.

The structure of the PseuP_222 and PseuP_224 genomes resembles that of lambdoid phages (Figure 3; Supplementary Datas S1 and S2). Following the genes of terminase small and large subunits, PseuP_222 and PseuP_224 genomes contain clusters of genes oriented in the forward direction, with most of them being the putative virion genes. In the clusters of both genomes, we identified the genes that encode portal protein, major capsid protein, and tail tube protein, as well as a conservative block of the lambdoid phage genes of tail tape measure protein and tail tip assembly proteins M, L, K, I, and J (central tip protein responsible for host specificity). Notably, the gene of a P5-like structural protein with alleged hydrolytic beta-N-acetylglucosaminidase activity was found in both genomes. Some genes from such clusters were either hypothetical or it was not possible to accurately identify the homologues in both genomes. The next clusters in the PseuP_222 and PseuP_224 genomes consist of the genes responsible for integration and DNA-processing enzymes. The genes of integrase and nucleoid-associated protein NdpA/YejK (oriented in the reverse direction) were found in such clusters of both phages; however, most genes from the clusters were hypothetical ones (Supplementary Datas S1 and S2). The subsequent clusters in the PseuP_222 and PseuP_224 genomes include the lysogeny and replication genes. The genes encoding the CI-like repressor and the antitermination protein flank such clusters in the phages. The PseuP_222 genome encodes replication protein O and replisome organizer, which participate in the replication fork formation, as well as the NinB and NinG recombination proteins. In the PseuP_224 genome, the gene of the site-specific phage integrase, and two genes encoding proteins with helicase activity, primosomal protein 1 and DnaB, were located in this gene cluster. The genome of PseuP_222 also contains the genes of Cro and CII proteins; however, it was not possible to identify homologs of such genes in the PseuP_224 genome. As for the lysis clusters, we found the gene of lambda family holin in the PseuP_224 genome. Notably, the precise determination of clusters in both genomes was difficult due to mosaicism and a large number of genes encoding hypothetical proteins (Figure 3).

Despite the fact that PseuP_222 and PseuP_224 belong to a group of lambdoid phages, their genomes show substantial divergence (Figure 3). Nucleotide identity between the complete genomes was calculated in the BioEdit 7.2.5 program [32] based on the alignment, which was performed in the MAFFT program (https://mafft.cbrc.jp accessed on 13 April 2023). The calculated nucleotide identity (NI) was low: 37.5%. This range is lower than the cut-off (70% NI of the complete genome length) established by the ICTV Bacterial Virus Subcommittee for creating phage genera [35]. Only a few genes from both genomes demonstrated high similarity between themselves: the genes encoding nucleoid-associated proteins NdpA/YejK (NI ~88%) and phage tail tip attachment proteins J (NI ~62%,), as well as the genes encoding P5-like structural proteins with putative hydrolytic activity (NI 89.7%) and the adjacent structural (NI 82.6%) and hypothetical (NI 82.6%) genes (Figure 3). Based on the substantial difference in the genome size, number of ORFs, and genome similarity, we can conclude that the PseuP_222 and PseuP_224 phages are not closely related, although they have the same host strain.

### 3.5. Comparative Analysis of the PseuP_222 and PseuP_224 Phages with Other Phages

**Figure 4:**
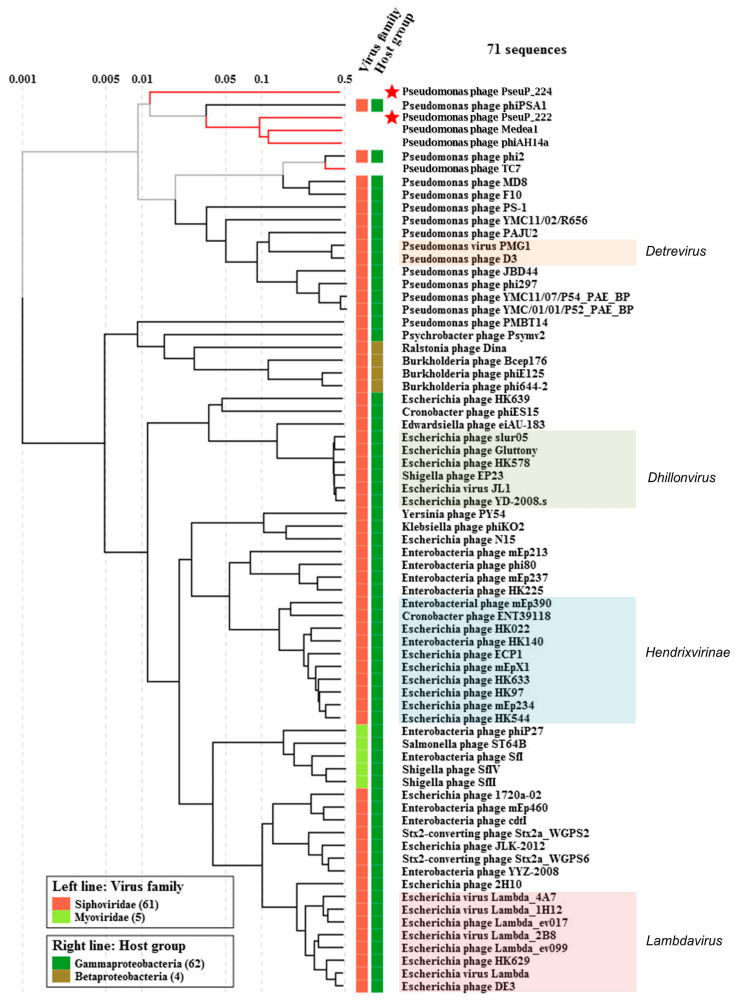
VipTree analysis of PseuP_222 and PseuP_224 phages. The studied phages PseuP_222 and PseuP_224 are marked with red stars. Phage sequences that were downloaded from the NCBI GenBank manually are marked with red phylogenetic branches. Phages with closely related sequences were manually removed, except for phages of environmental *Pseudomonas* spp.

Comparative ViPTree proteomic analysis shows that PseuP_222 and PseuP_224 with the *Pseudomonas* phages phiPSA1 [36], Medea1 (both *P. syringae* phages), and phiAH14a (Antarctic *Pseudomonas* sp.) [37] form a group of genetically divergent phages of environmental *Pseudomonas* species and that this group is distant from a large group of *P. aeruginosa* phages (Figure 4).

PseuP_222 clustered with the *Pseudomonas* phages phiAH14a and Medea1; however, substantial divergences between these three bacteriophages do not allow combining them into one genus (Figure 4). Further isolation and characterization of phages related to these three phages will probably provide accurate taxonomic classification of the phages and constitute new genus/genera. As for PseuP_224, its genome formed a separate branch, which is remote from all other phages of environmental *Pseudomonas* (Figure 4). Genetic and proteomic differences of PseuP_224 exceed those for other phages of environmental *Pseudomonas* spp.

**Figure 5:**
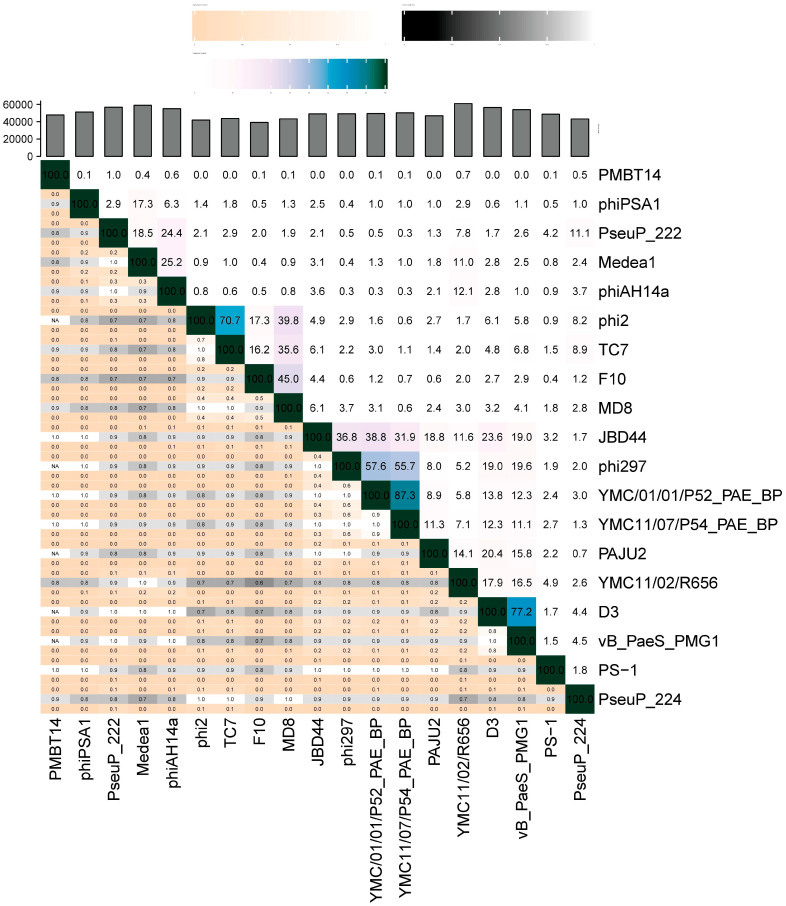
Matrix of intergenomic similarities calculated using VIRIDIC for PseuP_222, PseuP_224, and other *Pseudomonas* phages represented in Figure 4.

In addition, a matrix of intergenomic similarities of the *Pseudomonas* phage genomes was determined using VIRIDIC (Figure 5). Pairwise similarity of the genomes of environmental *Pseudomonas* phages (PseuP_222, PseuP_224, phiPSA1, Medea1, and phiAH14a) in all cases was significantly lower than the genus threshold of 70% [34] and did not exceed 25.6% (identity between Medea1 and phiAH14a) and 24.7% (identity between PseuP_222 and phiAH14a). Identity levels of most genomes of *P. aeruginosa* siphophages were higher than those for environmental *Pseudomonas* phages, and at least two new genera could be established in addition to the existing *Detrevirus* genus. *Pseudomonas* phages phi2 and TC7 could form one new genus, whereas phages phi297, YMC01/01/P52_PAE_BP, and YMC11/07/P54_PAE_BP could be members of another new genus (Figure 5).

### 3.6. Phylogenetic Analysis of the PseuP_222 and PseuP_224 Proteins

Phylogenetic trees of the PseuP_222 and PseuP_224 terminase large subunits, major capsid proteins, tail tape measure proteins, and CI-like repressors were constructed using appropriate sequences of *Pseudomonas* phages that were searched with BLASTP (E value < 0.05), as well as five bacterial and five viral most similar sequences from the GenBank database. In addition, annotated proteins similar to the corresponding proteins of studied PseuP_222 and PseuP_224 were downloaded from a database containing 17 *Pseudomonas* phages presented in the constructed ViPTree phylogeny (Figure 4) and manually added to the analysis. Corresponding sequences of two *Escherichia* lambda phages were manually added to the analysis.

**Figure 6:**
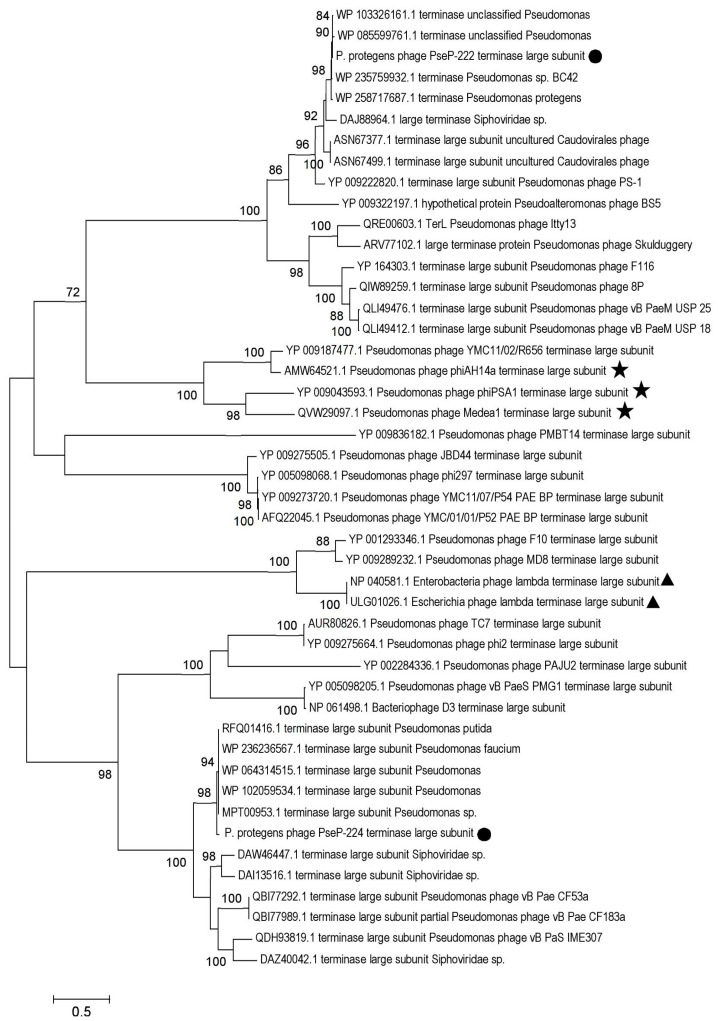
Maximum likelihood phylogenetic tree of the PseuP_222 and PseuP_224 terminase large subunits. Sequences of the studied phages PseuP_222 and PseuP_224 are marked with black circles, sequences of *Escherichia* phage lambda are marked with black triangles, and environmental *Pseudomonas* phage sequences are noted with black stars. Statistical support above 70% is shown at the nodes; 1000 bootstrap replications were applied.

Phylogenetic analysis of the terminase large subunits of PseuP_222 and PseuP_224 demonstrated that these proteins were positioned in distant clades (Figure 6). The PseuP_222 terminase large subunit was in a big clade containing individual well-supported branches with terminase sequences of environmental *Pseudomonas* phages (phiAH14a, Medea1, and phiPSA1) and *P. aeruginosa* phages. Notably, several terminase sequences from *Pseudomonas* phages with podovirus morphotype (8P, F116, Skulduggery) were found within the clade, which indicates complex evolution of phages with different morphology. Another big clade of terminase sequences contained PseuP_224 terminase large subunit and individual branches with those of *P. aeruginosa* phages, *Escherichia* phages lambda, and members of the *Detrevirus* genus. Since the terminase large subunit is a conservative protein and is often used for taxonomic determination [38], the constructed phylogenetic tree suggests a different evolution history for the studied PseuP_222 and PseuP_224 phages (Figure 6).

**Figure 7:**
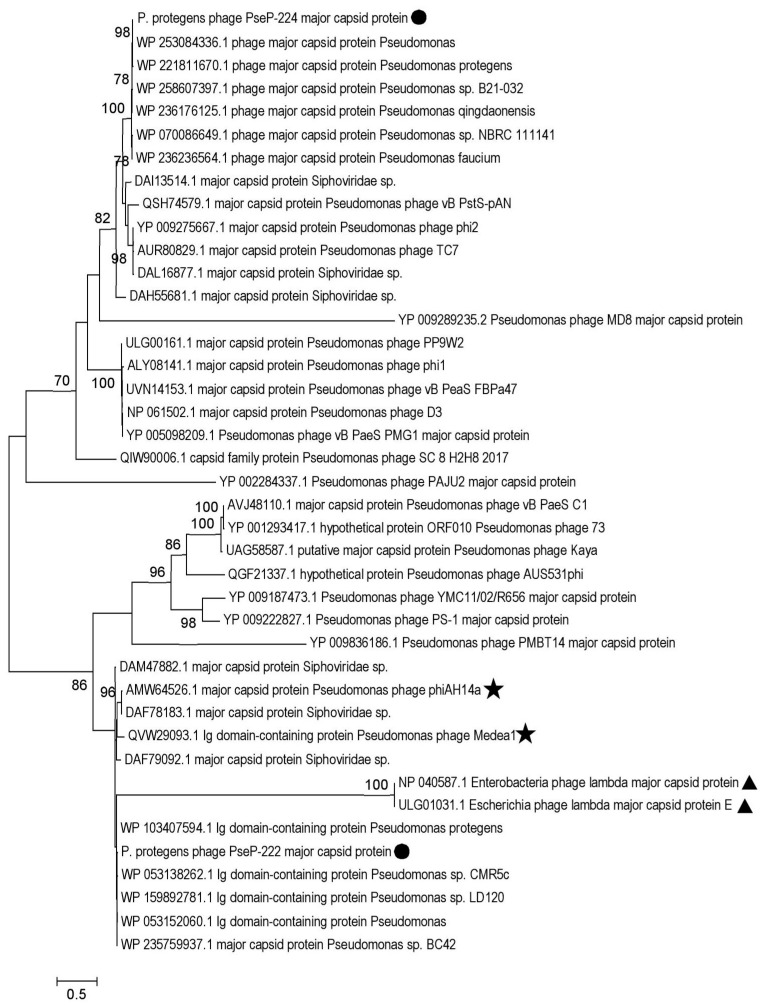
Maximum likelihood phylogenetic tree of the PseuP_222 and PseuP_224 major capsid protein. Sequences of the studied phages PseuP_222 and PseuP_224 are marked with black circles, sequences of *Escherichia* phage lambda are marked with black triangles, and environmental *Pseudomonas* phage sequences are noted with black stars. Statistical support above 70% is shown at the nodes; 1000 bootstrap replications were applied.

In the phylogenetic tree of major capsid protein sequences (Figure 7), PseuP_222 grouped with phages of environmental *Pseudomonas* (phiAH14a and Medea1) and they form a well-supported branch of closely related sequences with those of unclassified siphophages and *Pseudomonas* prophages. This branch was positioned in a large clade containing lambda phages, *P. aeruginosa* phage PMBT14, and a branch with *P. aeruginosa* phages with siphovirus morphology. As in the case of the terminase tree, PseuP_224 was in another clade distant from PseuP_222. Along with PseuP_224, other *P. aeruginosa* phages with siphovirus morphology form branches within this big clade (Figure 7).

The phylogenetic tree of tail tape-measure protein sequences reflected a similar trend as the phylogenetic trees of terminase and major capsid protein sequences (Figure S1). PseuP_222 and PseuP_224 sequences were positioned in distant clades. As in the phylogenetic trees described above, PseuP_222 was in a large clade including phages of both environmental *Pseudomonas* (phiAH14a and Medea1) and *P. aeruginosa*, whereas PseuP_224 was in another clade, which contains branches formed by *P. aeruginosa* phages with siphovirus morphology.

**Figure 8:**
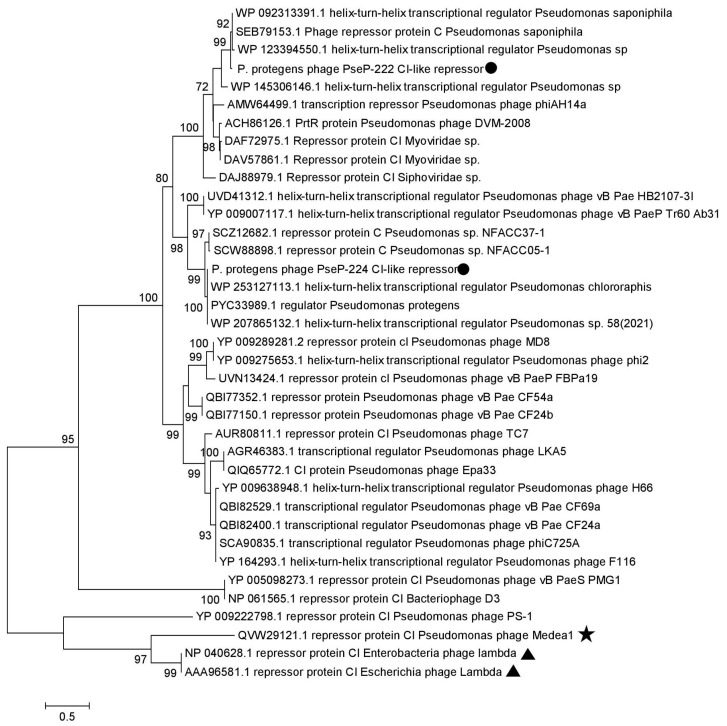
Maximum likelihood phylogenetic tree of the PseuP_222 and PseuP_224 C1-like repressor. Sequences of the studied phages PseuP_222 and PseuP_224 are marked with black circles, sequences of *Escherichia* phage lambda are marked with black triangles, and environmental *Pseudomonas* phage sequences are noted with black stars. Statistical support above 70% is shown at the nodes; 1000 bootstrap replications were applied.

As for the phylogenetic tree of CI repressor protein sequences (Figure 8), its topology differed from the trees described above (Figure 6, Figure 7 and Figure S1). The tree indicated a closer phylogenetic relationship between CI repressors of PseuP_222 and PseuP_224, as opposed to the terminases, major capsid proteins, and tail tape-measure proteins of the phages. Notably, CI repressor sequences of unclassified myophages and the phiAH14a phage of the environmental Antarctic *Pseudomonas* sp. grouped with PseuP_222 in the branch, whereas no sequences from known *Pseudomonas* phages related to PseuP_224 were identified in the branch with PseuP_224 CI repressor (Figure 8). Our phylogenetic analysis did not indicate close relationships between PseuP_222 and PseuP_224 CI repressor proteins and those of phage lambda.

In addition to the phylogeny of products of the signature genes, phylogenetic analysis of the PseuP_222 and PseuP_224 nucleoid-associated proteins NdpA/YejK and P5-like structural proteins with putative hydrolytic activity was performed. Constructed phylogenetic trees demonstrated close phylogenetic relations of NdpA/YejK proteins of both phages, as well as P5-like proteins (Supplementary Figures S2 and S3). In both trees, these phage proteins grouped into well-supported branches with similar bacterial/prophage sequences of environmental *Pseudomonas* spp. (in the case of NdpA/YejK proteins) and *P. protegens* (in the case of P5-like proteins).

Notably, endolysins from lambdoid *Pseudomonas* phages did not demonstrate evolutionary relationships with those of PseuP_222 and PseuP_224 (Figure S2). No homologs of NdpA/YejK proteins in the lambdoid *Pseudomonas* phages were found (Figure S3). The obtained results indicate that these two genes were possibly acquired by the PseuP_222 and PseuP_224 genomes during their coevolution with the host/group of hosts. Taking into consideration substantial divergence of the PseuP_222 and PseuP_224 genomes, unlike the similarity of the genes of NdpA/YejK and P5/endolysis, we can assume that this evolutionary event occurred relatively recently.

### 3.7. Comparative Analysis of the PseuP_222 and PseuP_224 Genomes with the Genomes of P. protegens Prophages

In most cases, phylogenetic analysis of PseuP_222 and PseuP_224 phage proteins showed their similarity to the corresponding bacterial/prophage proteins, so the genomes of both phages were compared with bacterial genomes extracted from the GenBank using BLASTN. PseuP_222 genome fragments demonstrated high nucleotide similarity with individual fragments of the genomes of *P. protegens* CHA0 (LS999205.1), B21-030 (CP087182.1), H78 (CP013184.1), and PPRAR02 (CP054872.1) (NI < 93%, coverage < 44%). For PseuP_224, the most similar sequence fragments were found in the genome of *P. protegens* PS1 (CP081490.1) (NI 95%, coverage 39%). In addition, fragments of the PseuP_224 genome demonstrated some similarity with those of the 36 kb prophage found in the genome of the reference strain *P. protegens* CHA0 (LS999205.1) using PHASTER software (https://phaster.ca accessed on 13 April 2023). Dot-blot analysis by MAFFT software is shown in Supplementary Figure S4, indicating very limited similarity between two genomes. The 36 kb prophage possibly occurs in many *P. protegens* strains, and its genome was found in the genomes of *P. protegens* PGNL1 (CP054874.1) and PGNR2 (CP054873.1) using BLASTN. The obtained data support the possibility of horizontal transfer of some genes into the PseuP_222 and PseuP_224 genomes.

## 4. Discussion

In this study, two novel *P. protegens* phages, PseuP_222 and PseuP_224, and their *P. protegens* 4060 host strain were isolated from the same water sample from the river Inya, Novosibirsk Province. Despite the fact that PseuP_222 and PseuP_224 used the same host, they produced plaques with different morphology and had different lytic properties. Electron microscopy and genome structure indicated that both phages have siphovirus morphology and belong to a group of lambdoid phages. Comparative analyses of the PseuP_222 and PseuP_224 genomes revealed that they substantially differ between themselves and from other available phage genomes, and calculated NIs are significantly lower than the genus threshold (70% NI of the complete genome length), established by ICTV Bacterial Virus Subcommitee for creating phage genera [34]. Proteomic analysis indicated that PseuP_222 and PseuP_224 are members of a group of genetically diverse siphophages of environmental *Pseudomonas* spp. and the studied phages are distant from a large group of *P. aeruginosa* phages.

Phylogenetic analysis of the terminase large subunits, major capsid proteins, and tail tape measure proteins confirmed substantial distance of the corresponding proteins between PseuP_222 and PseuP_224 and other *Pseudomonas* phages. In the phylogenetic trees, the positioning of the above proteins changed relative to corresponding proteins of other *Pseudomonas* phages with the siphovirus morphology, indicating mosaicism inherent for such phages [39,40,41,42]. In some cases, corresponding proteins of Podo- and Myoviridae were closer than Siphoviridae ones. Only CI-like repressors of the studied phages showed some similarity. However, the PseuP_222 CI-like repressor was still closer to those of Myoviridae phages than to the PseuP_224 CI-like repressor, and both repressors were distant from the lambda CI repressor. It should be noted that unlike the above proteins, nucleoid-associated proteins NdpA/YejK encoded by the PseuP_222 and PseuP_224 genomes demonstrated high similarity, as well as P5-like structural proteins with putative hydrolytic activity of both phages. The obtained results of phylogenetic analysis indicated that the evolutionary history of PseuP_222 and PseuP_224 was mostly independent and that these two phages probably began to use one host/similar hosts only recently, acquiring new genes whose products have not yet accumulated significant differences. Notably, the results on the multistep bacterial killing curves obtained with individual phages and their mixture indicated that there is possible competition of PseuP_222 and PseuP_224 for the cellular receptor. To test this assumption, the genes encoding tail tip proteins J in PseuP_222 and PseuP_224 were compared using dot-plot analysis (Supplementary Figure S5). The obtained result indicated that the 3′-part of the genes is very close. Taking into consideration that the C-part of the tail tip protein J in lambda phage is responsible for binding with the *E. coli* receptor, we can hypothesize that the high similarity of C-parts of the tail tip protein J in phages PseuP_222 and PseuP_224 confirms that both phages can recognize the same cell receptor on the surface of their *P. protegens* 4060 host strain.

Substantial divergences of the PseuP_222 and PseuP_224 genomes and proteomes between themselves, and between them and other described phages preclude us from proposing taxonomy of the phages. Since *P. protegens* strains from many environments remain still unstudied from a microbiological point of view, it can be expected that new *P. protegens* phages will be found and that thetaxonomy of phages of environmental *Pseudomonas* will be clarified. Considering that *P. protegens* strains can be used as soil improvers and natural insecticides, data on PseuP_222 and PseuP_224 and other environmental phages, including ΦGP100 [18,19], which can lyse potentially useful *P. protegens* strains or influence their antibacterial properties, should be considered.

## Data Availability

Not applicable.

## Supplementary Materials

The following supporting information can be downloaded at: https://www.mdpi.com/article/10.3390/microorganisms11061456/s1, Figure S1: Maximum likelihood phylogenetic tree of the PseuP_222 and PseuP_224 tail tape measure protein. Sequences of the studied phages PseuP_222 and PseuP_224 are marked with black circles, sequences of *Escherichia* phage lambda are marked with black triangles, and environmental *Pseudomonas* phage sequences are noted with stars. Statistical support above 70% is shown at the nodes; 1000 bootstrap replications were applied. Figure S2: Maximum likelihood phylogenetic tree of the PseuP_222 and PseuP_224 NdpA/YejK protein. Sequences of the studied phages PseuP_222 and PseuP_224 are marked with black circles, sequences of *Escherichia* phage lambda are marked with black triangles. Statistical support above 70% is shown at the nodes; 1000 bootstrap replications were applied. Figure S3: Maximum likelihood phylogenetic tree of the PseuP_222 and PseuP_224 P5-like/putative endolysin protein. Sequences of the studied phages PseuP_222 and PseuP_224 are marked with black circles, sequences of *Escherichia* phage lambda are marked with black triangles, and environmental *Pseudomonas* phage sequences are noted with stars. Statistical support above 70% is shown at the nodes; 1000 bootstrap replications were applied. Figure S4: Dot-plot illustrating low level of similarity between PseuP_224 and prophage 36kb genomes; alignment and dot-plot were carried out using MAFFT software. Figure S5: Dot-plot illustrating high level of similarity between genes, encoding tail tip proteins J from both PseuP_222 and PseuP_224 genomes; alignment and dot-plot were carried out using MAFFT software. Table S1: Primers for PCR. Table S2: *Pseudomonas* strains screened in host range assay. Data S1: Annotation of the PseuP_222 genome using RAST v.2.0. Data S2: Annotation of the PseuP_224 genome using RAST v.2.0.
