# Supplementary material for: Identification, Characterization, and Genome Analysis of Two Novel Temperate Pseudomonas protegens Phages PseuP_222 and PseuP_224
Source: Microorganisms. 2023 May 31;11(6):1456. doi: 10.3390/microorganisms11061456 (PMC10305613; doi:10.3390/microorganisms11061456)
Supplement: Supplementary file 1 [file microorganisms-11-01456-s001.zip › Table S1.pdf]

Table S1. Primers for PCR

Target genes	Primer sequences (5′ → 3′)	Annealing temperature	Reference
16S rRNA	8F: AGRGTTTGATCCTGGCTCA<br>1350R: 5`-GACGGGCGGTGTGTACAAG	55°C	[21]
*rpo*D	rpoDf: ACTTCCCTGGCACGGTTGACCA<br>rpoDr: TCGACATGCGACGGTTGATGTC	60°C	[22]
*gyr*B	gyrBf: TTCAGCTGGGACATCCTGGCCAA<br>gyrBr: TCGATCATCTTGCCGACRACCA	65°C	[22]
*fdx*A	*fdx*Af: TGCGTCGACGGAYAGACAC<br>*fdx*Ar: GTCGTCACCGACAACTGCATCAAG	65°C	[22]
