# Supplementary material for: Identification, Characterization, and Genome Analysis of Two Novel Temperate Pseudomonas protegens Phages PseuP_222 and PseuP_224
Source: Microorganisms. 2023 May 31;11(6):1456. doi: 10.3390/microorganisms11061456 (PMC10305613; doi:10.3390/microorganisms11061456)
Supplement: Supplementary file 1 [file microorganisms-11-01456-s001.zip › Table S2.pdf]

Table S2. *Pseudomonas* strains screened in host range assay

#	Species name	CEMTC #	GenBank accession #	PseP_222	PseP_224	Sample, geographical location
1	*P. protegens*	3668	MT040048			insect larvae, Novosibirsk Region
2		3669	MT040049			insect larvae, Novosibirsk Region
3		3758	OP541587			clinical sample, Novosibirsk
4		4060	ON838113	1.0×10^7	1.8×10^7	water, Inya river, Novosibirsk Region
5		4377	OP602247			pond water, Novosibirsk
6		4496	ON838131			water of a mineral waterfall with hydrogen sulfide, Sakhalin Island
7		4502	OP649444			pond water, Novosibirsk Region
8		4556	OP602256			water, Malinovskoe lake, Novosibirsk Region
9		4731	ON838132			water with hydrogen sulfide, Sakhalin Island
10		5980	OP649446		2.0×10^6	water, Novosibirsk Reservoir (Ob Sea), Novosibirsk Region
11	*P. aeruginosa*	38				ATCC strain 9027
12		671				soil from a poultry farm, Novosibirsk Region
13		1125	OP800156			geothermal water, Uzon volcano caldera, Kamchatka Peninsula
14		1589	OP800159			sewage, Novosibirsk
15		2273	OP602241			soil from a poultry farm, Kemerovo Region
16		3518				clinical sample, Novosibirsk
17		3525				clinical sample, Novosibirsk
18		3527				clinical sample, Novosibirsk
19		3532	OP541585			clinical sample, Novosibirsk
20		3536	OP541586			clinical sample, Novosibirsk
21		3623				clinical sample, Novosibirsk
22		3671	MT040050			insect larvae, Novosibirsk
23		3823	OP541588			clinical sample, Novosibirsk
24		3931	OP541589			clinical sample, Novosibirsk
25		3943	OP541591			clinical sample, Kemerovo
26		3962	OP541593			clinical sample, Novosibirsk
27		4147	OP541595			clinical sample, Novosibirsk
28		4174	OP649442			clinical sample, Novosibirsk
29		4180	OP541596			clinical sample, Novosibirsk
30		4196	OP602244			soil from a poultry farm, Novosibirsk Region
31		4622				clinical sample, Novosibirsk
32		4716				clinical sample, Novosibirsk
33		5095				clinical sample, Novosibirsk
34		5199	OP541599			clinical sample, Novosibirsk
35*	*P. alcaligenes*	4061	ON838114			water, Inya river, Novosibirsk Region
36	*P. alcaliphila*	4332	ON838118			water, Uryupino lake, Novosibirsk Region

37	*P. amygdali*	4483	OP649443	water from a mineral spring with hydrogen sulfide, Sakhalin Island
38	*P. azotoformans*	1853	OP602239	onion bulb, Novosibirsk
39		2046	OP602240	water from a spring, Novosibirsk Region
40		4494	OP602250	mineral springs waterfall with hydrogen sulfide, Sakhalin Island
41		4676	OP602260	pond silt, Novosibirsk Region
42		4986	OP602263	sawdust-soil compost, Krasnoyarsk Region
43	*P. brassicacearum*	4542	ON838134	soil, reclamation for 30 years, Kemerovo Region
44		4547	OP800165	soil, reclamation for 2 years, Kemerovo Region
45		4664	OP602259	soil, reclamation for 2 years, Kemerovo Region
46		5281	OP602266	park soil, Sakhalin Island
47		5298	OP602268	lawn soil, Sakhalin Island
48		6459	OP800178	forest soil, Kemerovo Region
49		6480	OP800179	potato field soil, Kemerovo Region
50	*P. canadensis*	4675		pond silt, Novosibirsk
51	*P. chengduensis*/"*P.*	1363	OP800158	lake water, Altai Region
52	*indoloxydans*"	4505	OP800164	pond silt, Novosibirsk
53	*P. extremaustralis*	4495	OP800163	water of a mineral spring with hydrogen sulfide, Sakhalin Island
54		5453	OP602275	water, Sema river, Altai Republic
55		5458	OP602276	silt of lake Geysernoe, Altai Republic
56	*P. extremorientalis*	3888	OP800160	soil of Uedinenia Island, Kara Sea
57		4493	OP800162	water of a mineral spring with hydrogen sulfide, Sakhalin Island
58		5477	OP800174	water, Krutikha river, Novosibirsk Region
59	*P. fluorescens*	42		rhizosphere
60		1239	OP602236	water, Fumarolnoye lake, Uzon volcano caldera, Kamchatka Peninsula
61		4536	OP602253	lake water, Novosibirsk Region
62	*P. frederiksbergensis*	4488	OP800161	soil near a mineral spring, Sakhalin Island
63		4670	OP800166	river water, Novosibirsk Region
64		4800	OP800168	agricultural soil, Volgograd Region
65		4994	OP800169	silt of Lake Geysernoe, Altai Republic
66		5252	OP800170	overburden dump, Republic of Sakha (Yakutia)
67		5451	OP800172	water, Sema river, Altai Republic
68		5455	OP800173	Silt, Geysernoe Lake, Altai Republic
69		5940	OP800176	water, Novosibirsk reservoir (Ob Sea), Novosibirsk Region

70		6501	OP800180	soil, Kemerovo Region
71	*P. germanica*	5598	OP800175	microbial mat, mineral spring with hydrogen sulfide, Sakhalin Island
72	*P. jessenii*	4640	ON838147	water, Chemal river, Altai Republic
73*	*P. khazarica*	4063		water, Inya river, Novosibirsk Region
74	*P. kilonensis*	4783	OP800167	natural pasture soil, Stavropol Region
75	*P. koreensis*	1236	OP800157	geothermal water, Uzon volcano caldera, Kamchatka Peninsula
76		6502	OP800181	soil, Kemerovo Region
77		6543	OP800184	pond silt, Lipetsk Region
78	*P. mandelii*	1488	OP602237	underground cold storage in the permafrost, Republic of Sakha (Yakutia)
79		4522	OP602252	pond water, Novosibirsk
80		4557	OP602257	water, Malinovskoe lake, Novosibirsk Region
81		4996	OP602264	water, Geysernoe Lake, Altai Republic
82	*P. phaseolicola*			strain ATCC 21781
83	*P. resinovorans*	6563	OP800185	water, Psakho river, Sochi
84	*Pseudomonas sp. (P. edaphica)*	4489	OP602249	bottom sediments of a mineral spring, Sakhalin Island
85	*Pseudomonas sp. (P. endophytica)*	4577	OP602258	pond silt, Novosibirsk Region
86	*Pseudomonas sp. (P. grimontii*	4833	OP602261	swampy soil, Chany lake, Novosibirsk Region
87	*P. savastanoi*	4361	OP602246	bottom sediments of a mineral spring with hydrogen sulfide, Sakhalin Island
88	*P. syringae group*	4541	OP602254	fresh dumps of coal mines, Kemerovo Region
89	*P. thivervalensis*	4477	OP602248	bottom sediments of a mineral spring with hydrogen sulfide, Sakhalin Island
90		5078	OP649445	steppe soil, Stavropol Region
91	*P. veronii*	5286	OP800171	water, Deputatka stream, Republic of Sakha (Yakutia)
92		5952	OP800177	water of a stream, Primorsky Region
93		6513	OP800182	water, Sema river, Altai Republic
94		6527	OP800183	water of a stream, Primorsky Region
95		6693	OP800186	water, Staroe lake, Vladivostok
