# Supplementary figures and images for: Identification, Characterization, and Genome Analysis of Two Novel Temperate Pseudomonas protegens Phages PseuP_222 and PseuP_224
Source: Microorganisms. 2023 May 31;11(6):1456. doi: 10.3390/microorganisms11061456 (PMC10305613; doi:10.3390/microorganisms11061456)

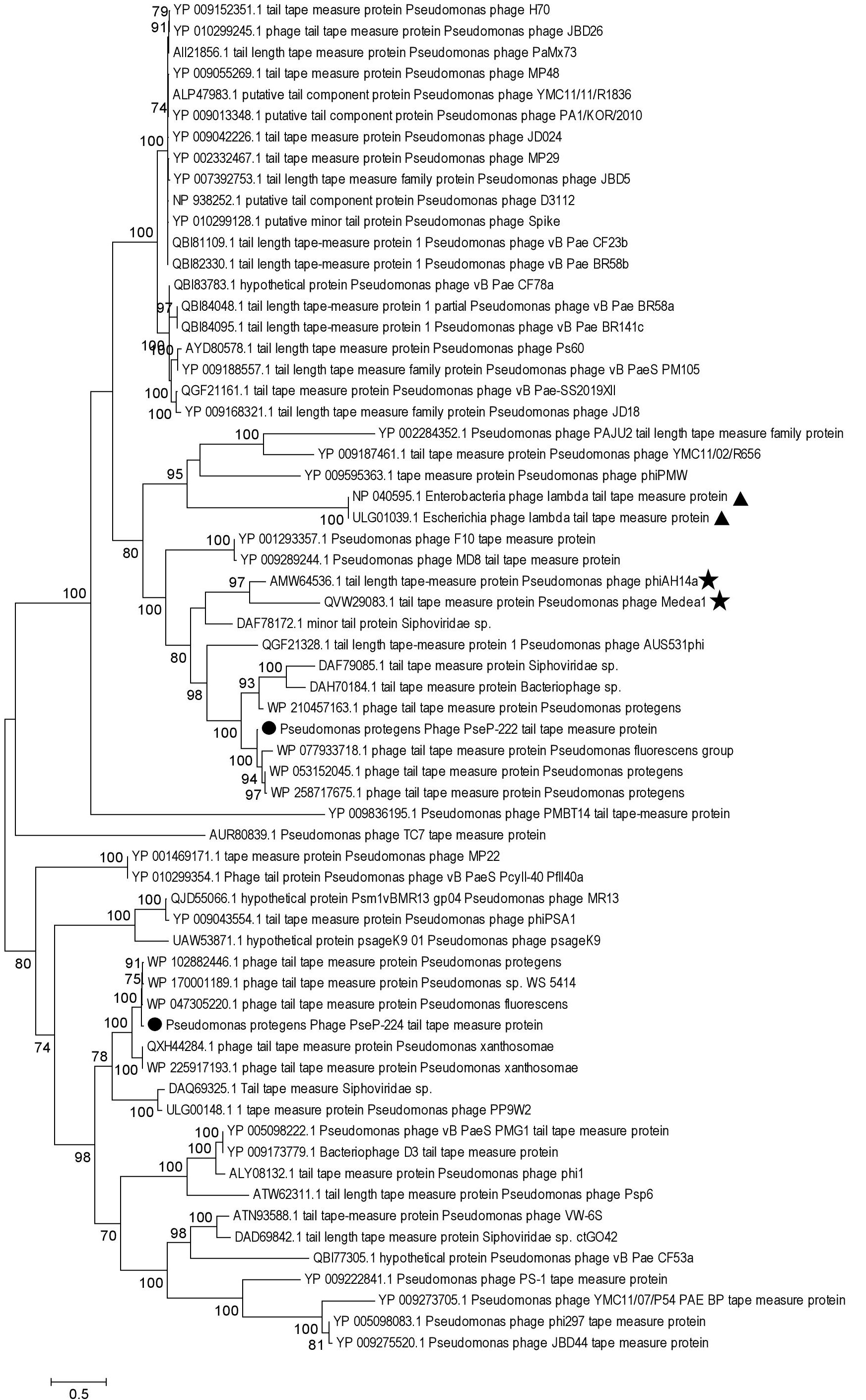

Supplement: Supplementary file 1 [file microorganisms-11-01456-s001.zip › Fig S1.jpg]

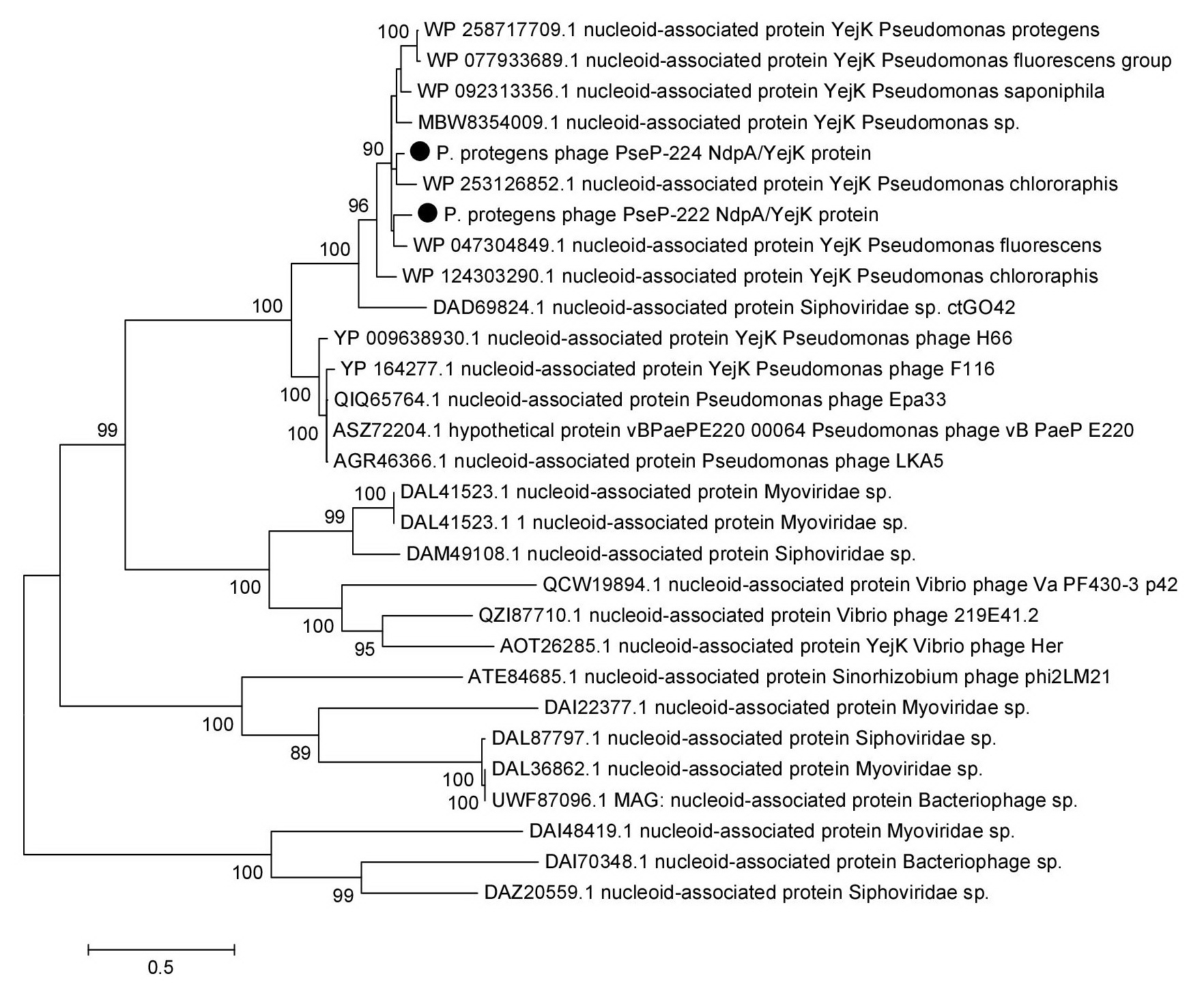

Supplement: Supplementary file 1 [file microorganisms-11-01456-s001.zip › Fig S2.jpg]

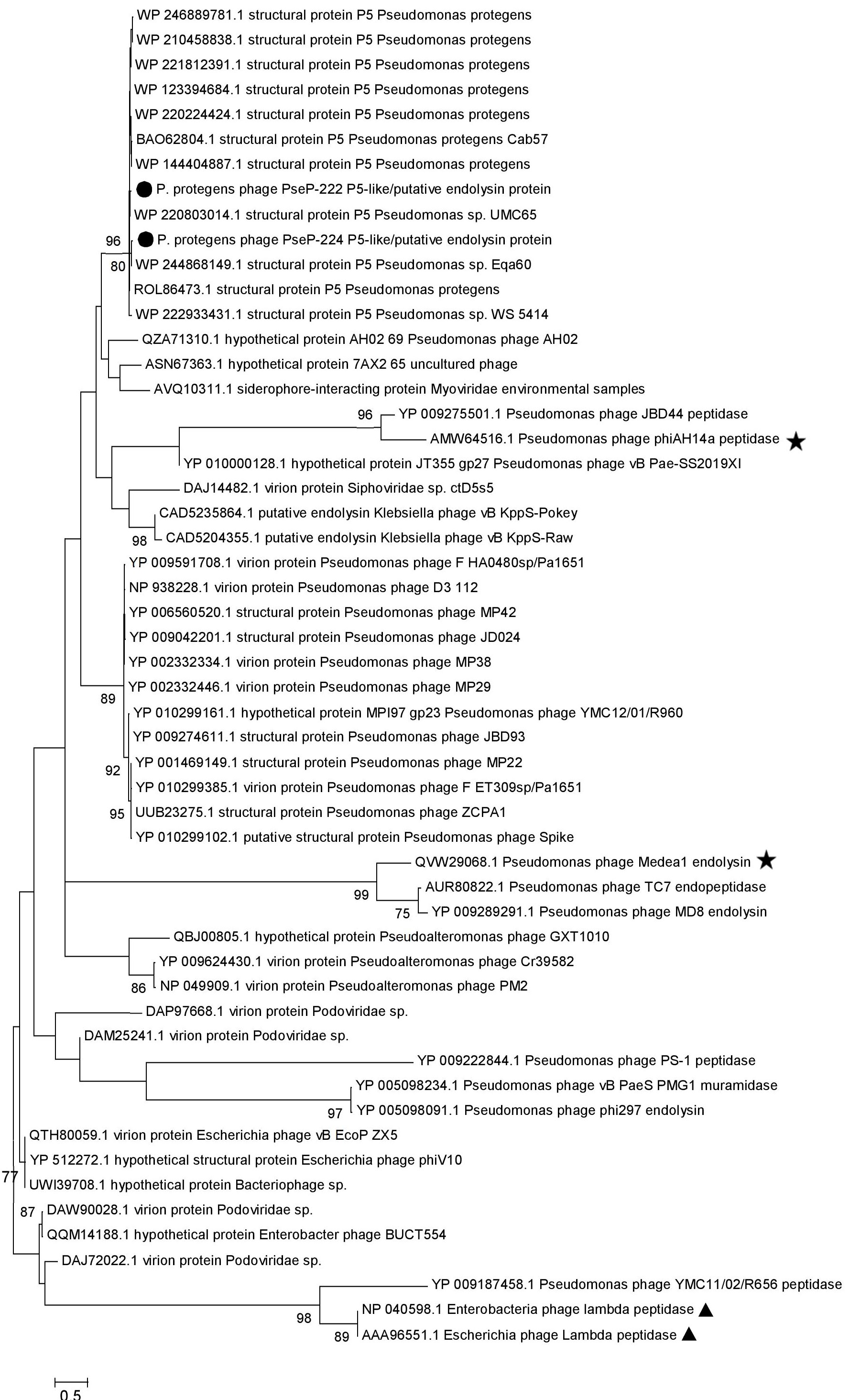

Supplement: Supplementary file 1 [file microorganisms-11-01456-s001.zip › Fig S3.jpg]

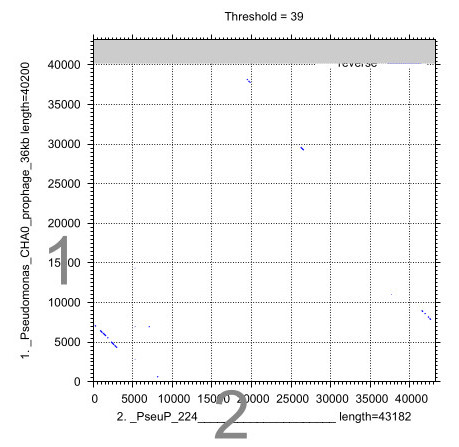

Supplement: Supplementary file 1 [file microorganisms-11-01456-s001.zip › Fig S4.jpg]

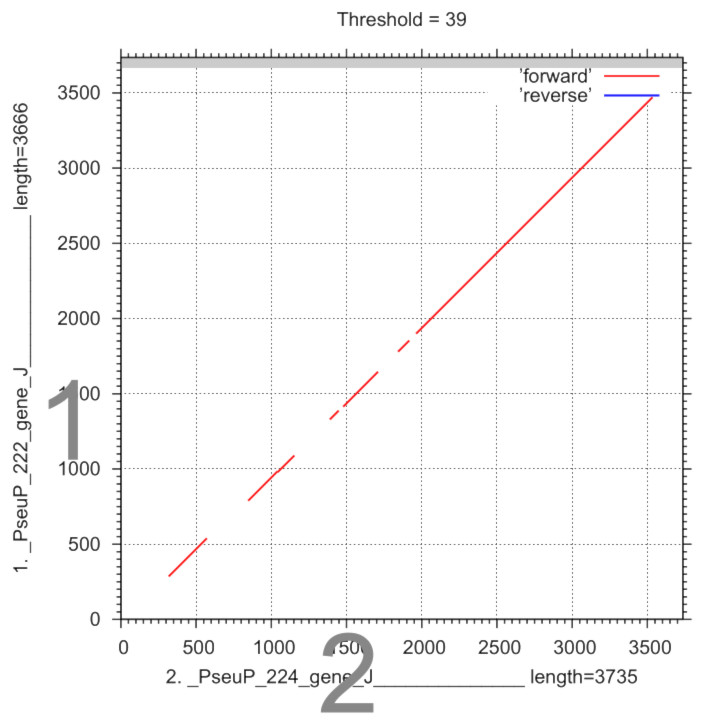

Supplement: Supplementary file 1 [file microorganisms-11-01456-s001.zip › Fig S5.jpg]
